# Safety and Efficacy of Salvage Neck Dissection Following Carbon-ion Radiotherapy with Chemotherapy for a Patient with Mucosal Malignant Melanoma of Head and Neck

**DOI:** 10.3390/diagnostics10020082

**Published:** 2020-02-03

**Authors:** Hidenori Suzuki, Eiichi Sasaki, Risa Motai, Seiya Goto, Daisuke Nishikawa, Shintaro Beppu, Hoshino Terada, Michi Sawabe, Nobuhiro Hanai

**Affiliations:** 1 Department of Head and Neck Surgery, Aichi Cancer Center Hospital, Nagoya 464-0021, Japan; rmotai@aichi-cc.jp (R.M.); s.goto@aichi-cc.jp (S.G.); dsknishi@aichi-cc.jp (D.N.); ncu.beppin3@gmail.com (S.B.); hoshinoterada@aichi-cc.jp (H.T.); m.sawabe@aichi-cc.jp (M.S.); hanai@aichi-cc.jp (N.H.); 2 Department of Pathology and Molecular Diagnostics, Aichi Cancer Center Hospital, Nagoya 464-0021, Japan; esasaki@aichi-cc.jp

**Keywords:** mucosal malignant melanoma in head and neck, salvage neck dissection, carbon ion radiotherapy, chemotherapy

## Abstract

Mucosal malignant melanoma of the head and neck is a rare diagnosis. The safety and efficacy of salvage neck dissection following carbon–ion radiotherapy with concurrent chemotherapy are not well described, and carbon–ion radiation protocols have not been fully developed. A 77 year old woman with crT0N1M0 mucosal melanoma of the head and neck achieved a complete response following initial treatment with carbon–ion radiotherapy and concurrent chemotherapy. She was treated with salvage neck dissection for as a cervical lymph node metastasis 16 months after initial treatment. She experienced neither Clavien-Dindo Grade 3 or 4 postoperative complications nor subsequent recurrence of disease at 3 months following salvage neck dissection. Surgical specimens may be useful for future precision oncology based on the molecular biology of recurrence melanoma with poor prognosis.

## 1. Introduction

Mucosal malignant melanoma of the head and neck (MMMHN) is very rare, accounting for less than 1% of all melanomas [1]. The prognosis following surgery with or without adjuvant radiotherapy is poor because of local and regional recurrences and distant metastasis [1]. Carbon–ion radiotherapy (CIR) with or without chemotherapy for MMMHN is expected as a high local control treatment [2]. Salvage neck dissection (ND) for regional recurrence following CIR with or without chemotherapy for MMMHN has been described [3,4]. Few reports of the safety and efficacy of salvage ND following CIR with chemotherapy for MMMHN included a description of any postoperative complications that occurred. The safety and efficacy of salvage ND following CIR plus chemotherapy in this patient with MMMHN adds to our knowledge of how to improve the prognosis of this rare condition.

## 2. Case Report

**Figure 1:**
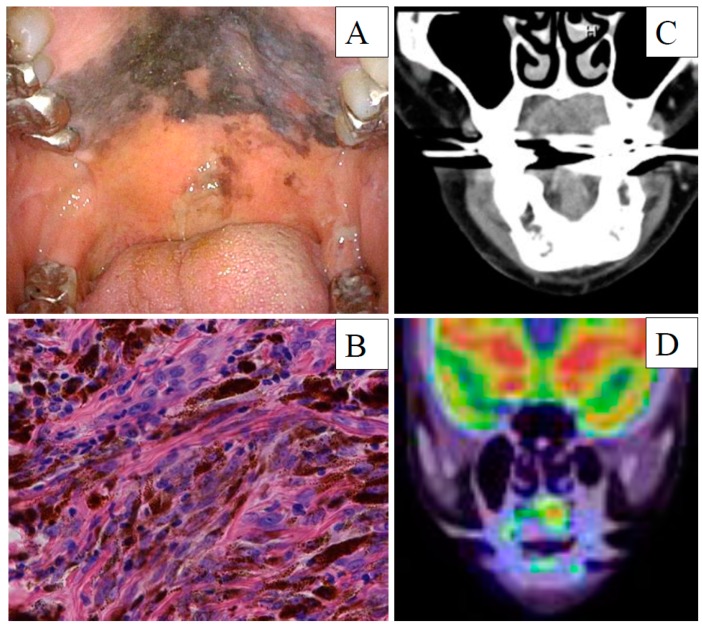
Mucosal malignant melanoma at the initial diagnosis. (**A**) White light endoscopy image, (**B**) Hematoxylin and eosin staining (**C**) Enhanced computed tomography image, and (**D**) 18F-fluorodeoxyglucose positron emission tomography with computed tomography.

**Figure 2:**
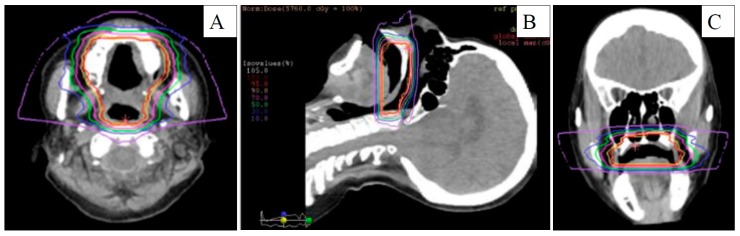
Computed tomography for carbon-ion dose distribution in initial mucosal melanoma. (**A**) Axial, (**B**) sagittal, and (**C**) coronal views. 70% dose: red-purple line, 50% dose: blue line, and purple line: 10%.

**Figure 3:**
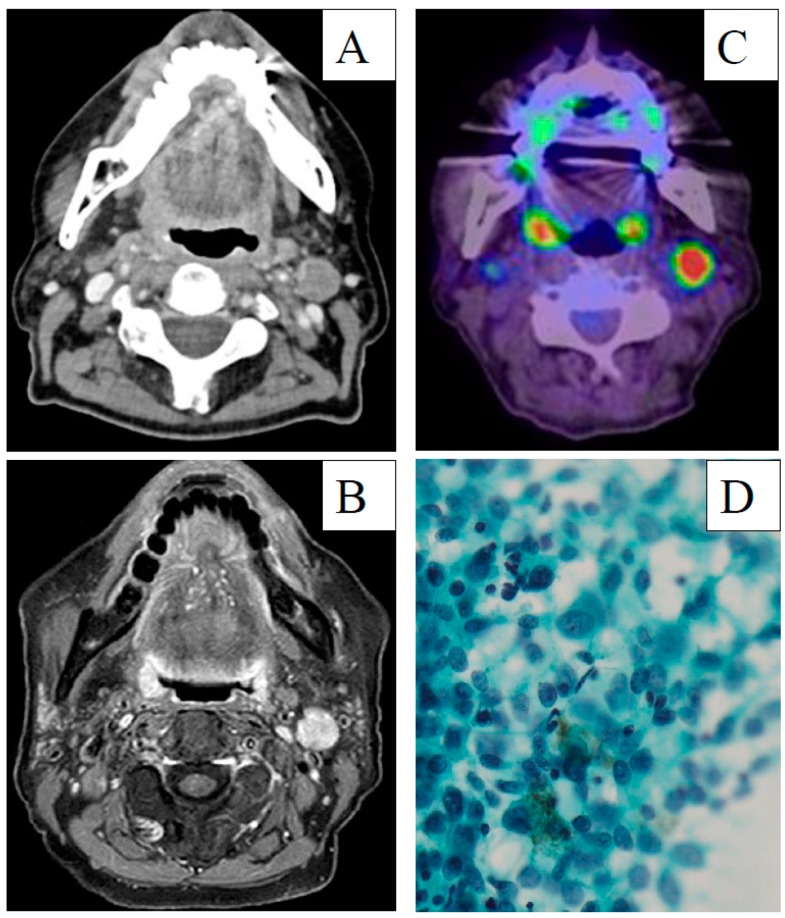
(**A**) Enhanced computed tomography image, (**B**) Magnetic resonance, (**C**) 18F-fluorodeoxyglucose positron emission tomography with computed tomography, imaged and (**D**) Papanicolaou staining of the metastatic lymph node. Scale bar = 100 µm.

A 77 year old woman with a 5 year history of mucosal melanosis of the hard palate was referred following diagnosis at another hospital of pathologically confirmed mucosal malignant melanoma. Visual inspection and endoscopy revealed that the oral tumor had spread extensively within the hard palate without involving the nasal cavity or nasopharynx. No neck lymph nodes were palpable. Enhanced and plain computed tomography (CT) and magnetic resonance imaging (MRI) revealed a 26 × 10 mm oral tumor without bone invasion or neck lymph node metastasis. ^18^F-fluorodeoxyglucose positron emission tomography with CT (^18^FDG-PET/CT) from the head to the thighs revealed abnormal uptake of the palate without any neck lesions. The diagnosis was stage of cT3N0M0 MMMHN, based on the eighth edition of the American Joint Committee on Cancer Staging Manual (Figure 1). The patient refused surgery and chose CIR at another institution as the initial treatment. CIR, with a total dose of 57.6 gray equivalents in 16 fractions, was administered for the oral tumor, with the dose distribution based on a planning CT (Figure 2). Concurrent chemotherapy included one cycle of 160 mg/body dacarbazine on days 1–5, 90 mg/body nimustine hydrocholoride on Day 1, and 0.9 mg/body vincristine on Day 1. The chemotherapy was stopped after one cycle because of severe hematological toxicity. The patient achieved a complete response following CIR and was followed-up at the outpatient clinic by physical examinations, endoscopy, enhanced CT, and MRI. At 16 months after CIR, enhanced CT and MRI detected a cervical metastasis of a left jugular lymph node with a maximum diameter of 17 mm and central necrosis. On ^18^FDG-PET/CT, the maximum standardized uptake value of the mass was 21.03. Echo-guided cytology of the lymph node revealed metastatic malignant melanoma. Biopsy of the mucosa melanosis of the hard palate revealed no evidence of malignancy. The recurrence disease was diagnosed as stage crT0N1M0 MMMHN (Figure 3).

**Figure 4:**
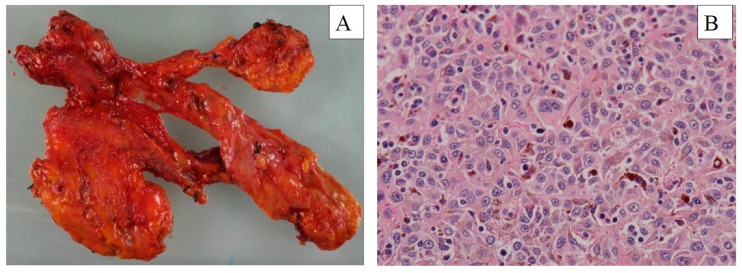
(**A**) Surgical specimen and (**B**) hematoxylin and eosin stained tissue following salvage neck dissection of the metastatic malignant melanoma. Scale bar = 100 µm. Arrows showed brown melanin. Please define the different color area in (**B**).

**Figure 5:**
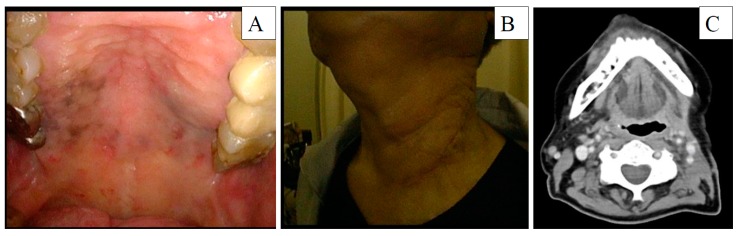
(**A**) White light images of the palate and (**B**) left neck and (**C**) enhanced computed tomography at 3 months after salvage neck dissection.

After considering radiotherapy and systemic immunotherapy, the patient was treated by en bloc salvage ND with preservation of the accessory nerve, internal jugular vein, and sternocleidomastoid muscle, as described by the Japan Neck Dissection Study Group [5]. Cefazolin (1 g) was administered as an antibiotic prophylaxis. The duration of surgery was 132 min and blood loss was less than 10 mL. Metastatic melanoma was pathologically confirmed in one of the 63 excised lymph nodes (Figure 4). The patient was discharged on postoperative Day 7. She experienced no Clavien-Dindo grade 3 or 4 postoperative complications. No recurrence was found by a whole-body enhanced CT performed 3 months after surgery (Figure 5). Written informed consent for the case report was obtained from the patient.

## 3. Discussion

The poor survival outcomes of MMMHN highlight the need more effective treatment [1,2]. Indeed, 5 year overall survival in a recent series of 25 MMMHN patients treated by surgery with or without adjuvant radiotherapy was 45.3% [6]. The Bragg peak characteristics of CIR offer a high relative biological effectiveness compared with conventional radiotherapy techniques, along with good local control and overall survival, and it would be expected to be effective in radioresistant MMMHN tumors [2,3,4]. Existing evidence supports the effectiveness of CIR for local control of MMMHN and related tumors. The safety of these treatments appears to be acceptable, but had not been fully described. Two of 32 patients with sinonasal mucosal melanoma included in a phase 2 study of CIR underwent salvage ND, but postoperative complications were not described [3]. A retrospective study of 74 patients with oral non-squamous cell carcinoma did not mention complications following salvage ND performed in eight patients [4]. Salvage surgery following CIR is a developing technique, and careful monitoring of postoperative complications with reporting of Clavien-Dindo or other complication scoring results is needed [7,8]. The outcomes achieved in this patient are in line with those reported by Naganawa et al. in a series of 19 patients of oral malignant melanoma treated by CIR alone, and three patients who required salvage treatments for regional recurrence did not develop re-recurrence until the last follow-up [9].

Additional data on postoperative complications of salvage surgery of local recurrence following CIR are available for several types of malignant tumors [7,8,10]. Salvage surgery with microsurgical reconstruction following CIR in a patient with osteosarcoma of the skull base resulted in uneventful wound healing without postoperative complications [10]. Salvage lobectomy with mediastinal node dissection in six lung cancer patients following CIR was safe and effective without severe complications [7]. Salvage surgery for local recurrence following CIR in 12 lung cancer patients was not associated with either mortality or Clavien-Dindo grade 3–4 complications [8]. We believe that the experience with this and other patients suggests that the safety of salvage ND for regional recurrence following CIR may be related to radiation dose [7,8,9,10]. Additional experience with rare cases of salvage ND in MMMHN after CIR with chemotherapy is needed for a more detailed evaluation of postoperative complications. Moreover, surgical specimens of recurrent MMMHN with poor prognosis may be useful for future precision oncology based on the molecular biology of melanoma.

## 4. Conclusions

A patient with stage crT0N1M0 MMMHN who underwent salvage ND after CIR with concurrent chemotherapy experienced neither grade 3–4 of Clavien-Dindo postoperative complications nor recurrence at 3 months of follow-up. Molecular biology from surgical specimens of recurrence MMMHN may be useful for future precision oncology.
